# Intra- and extra-uterine diagnosis and treatment of peripartum imperforate hymen: a case report

**DOI:** 10.3389/fped.2025.1546721

**Published:** 2025-03-31

**Authors:** Wei Bian, Jianmei Gu, Juan Yang, Yu Wang

**Affiliations:** ^1^ Department of Radiology, Jiaxing Maternity and Children Health Care Hospital, Affiliated Women and Children Hospital Jiaxing University, Jiaxing, China; ^2^ Department of Fetal Medicine Center, Jiaxing Maternity and Children Health Care Hospital, Affiliated Women and Children Hospital Jiaxing University, Jiaxing, China; ^3^ Department of Ultrasound, Jiaxing Maternity and Children Health Care Hospital, Affiliated Women and Children Hospital Jiaxing University, Jiaxing, China

**Keywords:** imperforate hymen, fetus, ultrasonography, magnetic resonance imaging, prenatal

## Abstract

The article reports a case of fetal imperforate hymen diagnosed by prenatal MRI and confirmed by postnatal surgery. The pregnant woman underwent routine 37-week ultrasound examination, which revealed an enlarged fetal bladder and an abdominal cystic mass. Subsequent 37-week + 6 prenatal MRI showed fetal uterine and vaginal fluid accumulation, suggesting congenital vaginal atresia. At 39 weeks + 5, under ultrasound guidance, amniotic, fetal abdominal, and vaginal cyst fluid were aspirated through a maternal abdominal puncture, avoiding the bowel. The cysts were significantly reduced in size, and the patient delivered vaginally at 39 weeks + 6. On postpartum day 1, pelvic MRI of the neonate showed normal uterine and cervical anatomy, with a lengthened, thickened vaginal wall and no fluid collection. On postpartum day 2, the patient underwent surgical exploration, which confirmed the diagnosis of imperforate hymen. Partial hymenectomy was performed without complication.

## Introduction

Congenital imperforate hymen is a common congenital malformation of the female reproductive tract. It typically results from incomplete canalization of the urogenital sinus during fetal development, leading to failure of the hymen to fully perforate (1). Imperforate hymen often presents with symptoms such as primary amenorrhea, gradually worsening cyclic lower abdominal pain, and an enlarging lower abdominal mass during puberty. Severe cases may also be accompanied by a feeling of rectal fullness, constipation, urinary frequency, or urinary retention (2, 3).

Ultrasound is the main diagnostic method for prenatal diagnosis of fetal hymenal atresia, but it is easily affected by factors such as maternal and fetal position. Application of MRI in this case can help guide treatment for prenatal and postnatal patients with Imperforate Hymen. The maternal was informed that her case would be submitted for publication and provided consent.

## Case report

**Figure 1:**
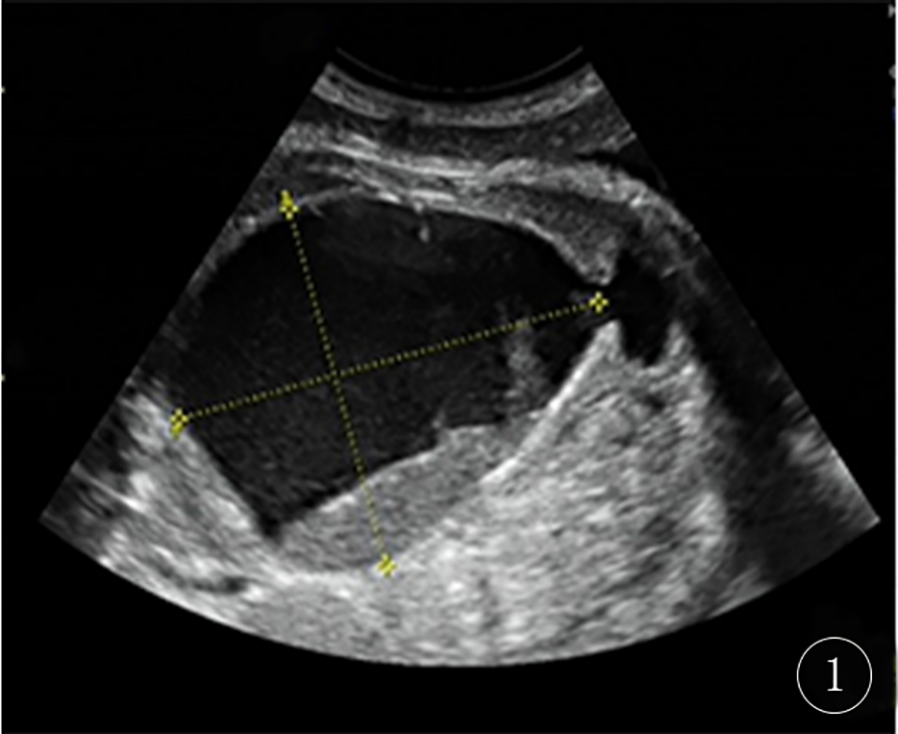
Fetal ultrasound at 37 weeks + 3 days the image shows a large cystic mass, approximately 64 mm × 83 mm in size, within the fetal pelvis. The cyst contents exhibit heterogeneous echogenicity.

A 29-year-old gravida 0, para 0, female patient at 37 weeks + 3 days (October 23, 2024) gestation presented to the Jiaxing Maternal and Child Health Care Hospital for routine prenatal ultrasound examination. The prenatal ultrasound revealed that the fetal bladder measured 4 mm × 64 mm × 83 mm (Figure 1), with internal septations and echogenic debris. The posterior wall of the bladder showed a 40 mm × 11 mm heterogeneous, slightly hyperechoic area. Bilateral umbilical arteries were visualized, and a 47 mm × 37 mm × 44 mm anechoic area was noted anterior to the left side of the bladder. No dilatation of the renal pelves or ureters was observed.

**Figure 2:**
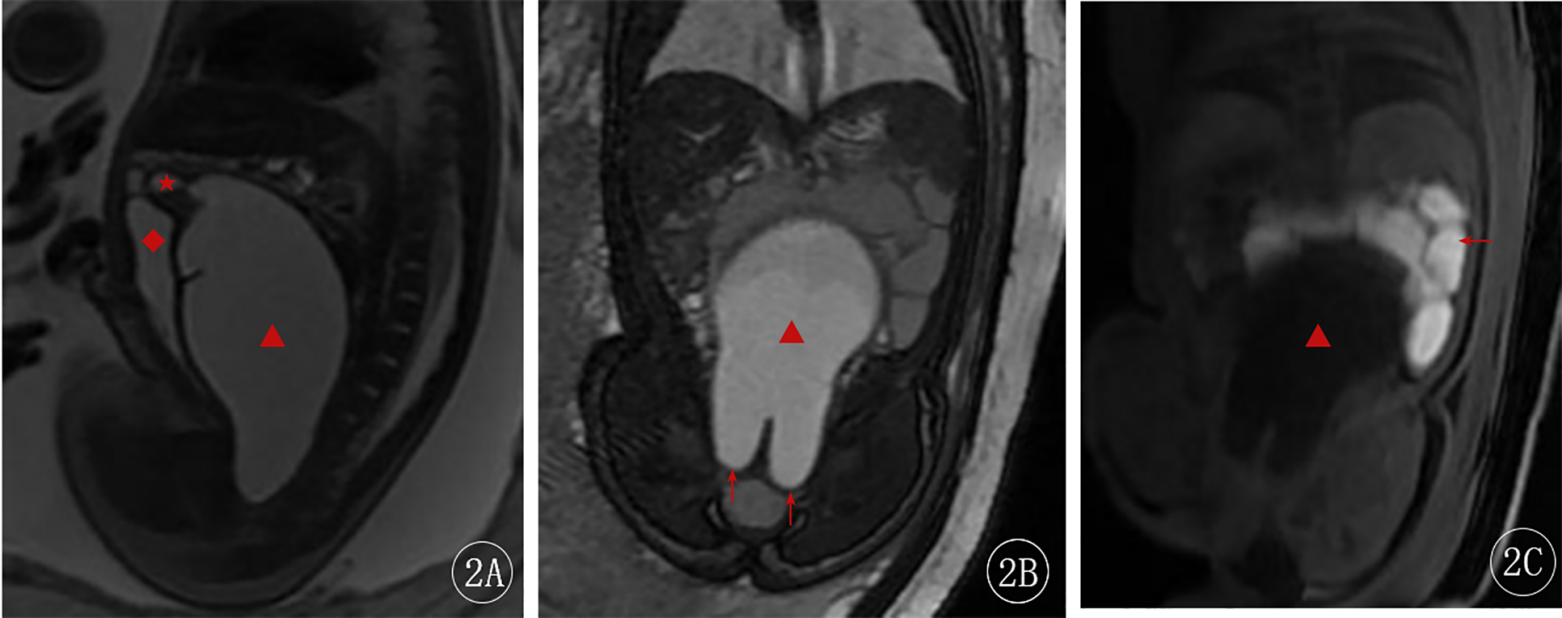
Fetal MRI at 37 weeks + 6 days. **(A)** Sagittal 2D FIESTA image—the red star (★) indicates the uterine cavity, and the red diamond (◆) shows the compressed bladder. **(B)** Coronal 2D FIESTA image—the red arrow points to the distal vaginal obstruction, showing the “reverse rabbit ear” sign. **(C)** Coronal LAVA-Flex image—the red arrow indicates the compressed bowel.

The ultrasound diagnosis was fetal bladder enlargement with an associated abdominal cystic mass. At 37 weeks + 6 days, a fetal MRI was performed, which showed a cystic structure measuring approximately 100 mm × 50 mm × 57 mm posterior to the fetal bladder. The cyst had low signal on T1-weighted images and high signal on T2-weighted images, with heterogeneous internal signal. The inferior margin of the cyst showed a “reverse rabbit ear” sign. A uterus-like structure was connected to the superior aspect of the cyst, with a small amount of fluid within its cavity, measuring approximately 10 mm × 6 mm. Compression of the adjacent bowel and bladder was noted (Figure 2). The fetal MRI diagnosis was a fetal abdominal cystic structure, most likely representing an imperforate hymen with associated voluminous vaginal and uterine fluid collection, as well as localized vaginal septation or adhesions in the superior and inferior aspects of the vagina.

At 39 weeks + 5 days, under ultrasound guidance, a 20G spinal needle was inserted through the maternal abdominal wall, into the amniotic cavity, and then into the fetal abdominal cavity and vaginal cyst (avoiding the bowel). Turbid fluid was aspirated. Post-aspiration ultrasound showed significant reduction in the size of the cyst. The needle was removed, and the puncture site was covered with a dressing. Fetal heart rate remained stable at 138 bpm.

The cyst fluid cytology revealed numerous mature squamous epithelial cells. At 39 weeks + 6 days, the patient had a spontaneous vaginal delivery of a female neonate weighing 3,400 g, with an Apgar score of 10. The placenta was delivered spontaneously and intact. The patient had a first-degree perineal laceration that was repaired.

**Figure 3:**
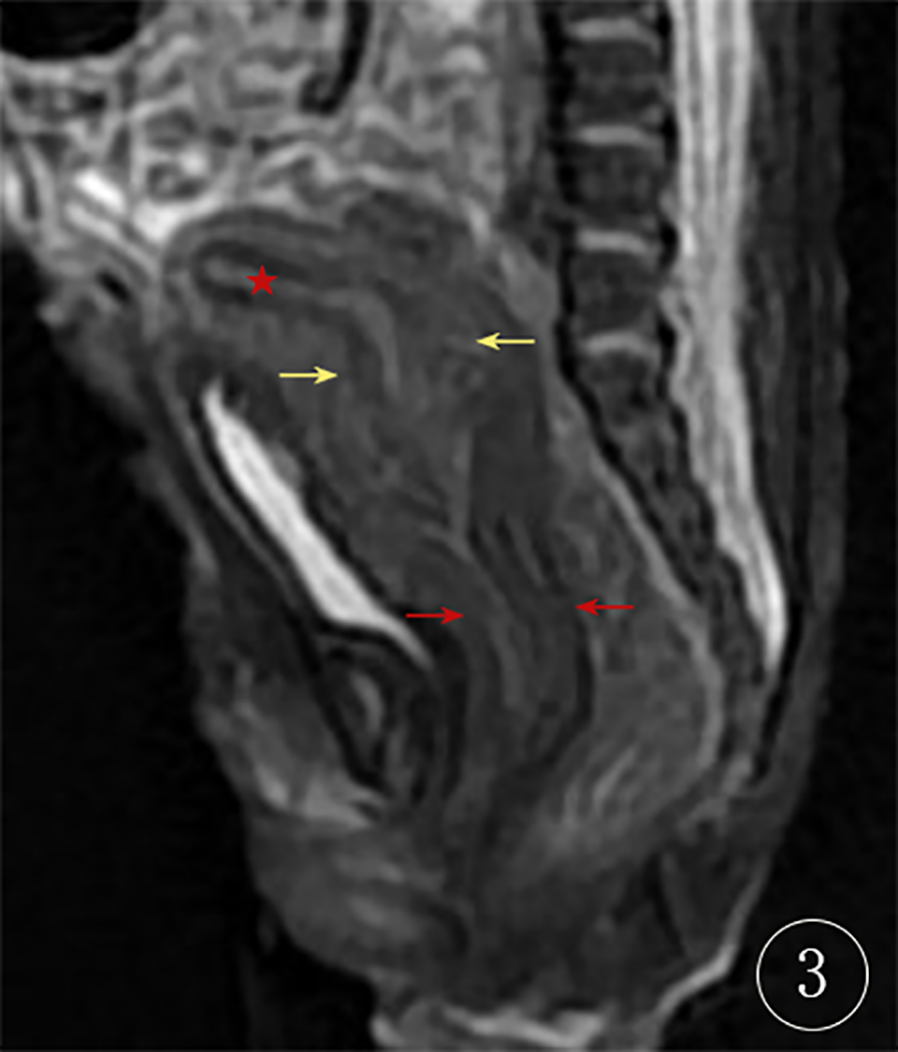
Neonatal MRI at 1 day postpartum the red star (★) represents the uterine cavity, the yellow arrow indicates the cervix, and the red arrow points to the thickened vaginal wall.

**Figure 4:**
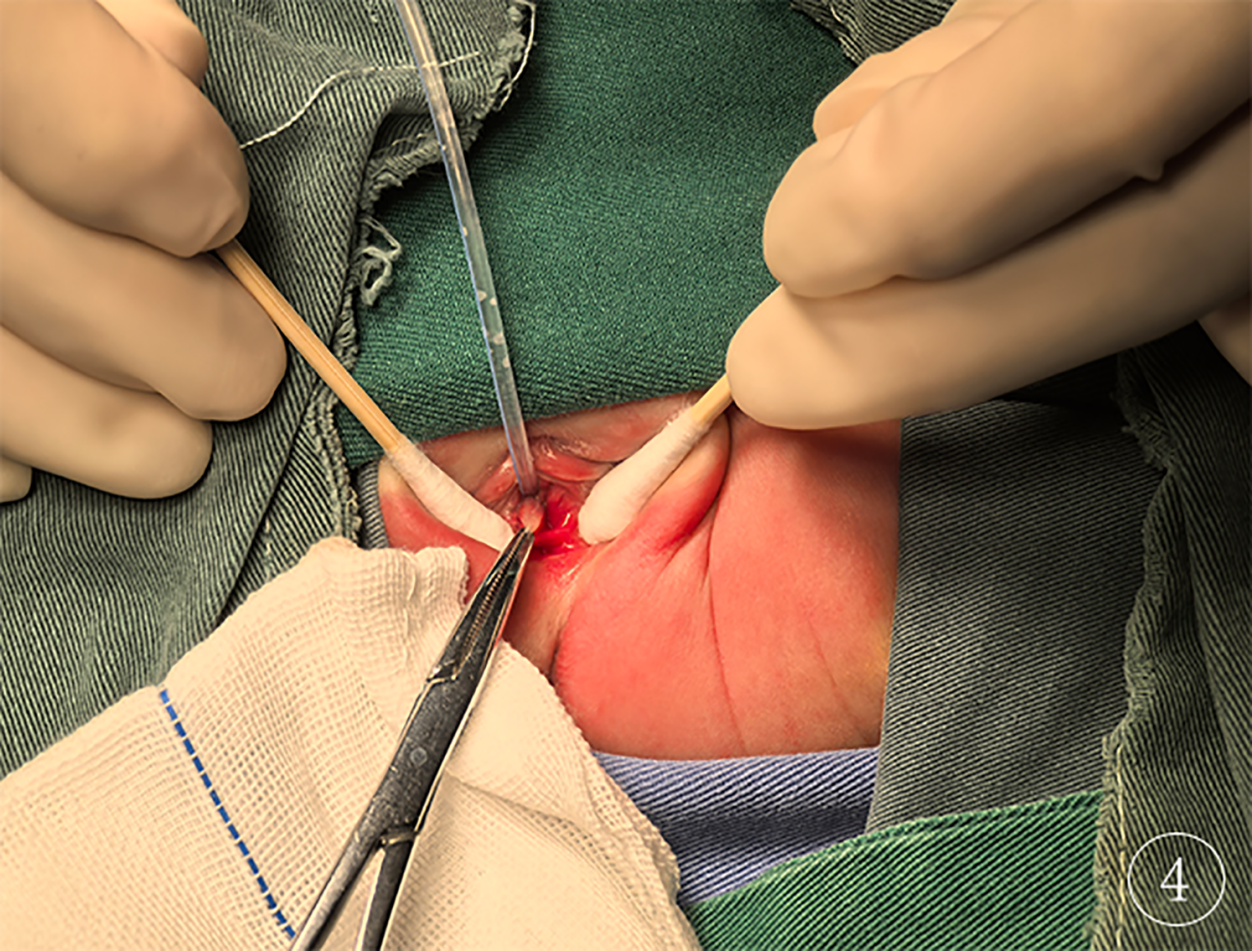
Intraoperative findings the image clearly demonstrates the diagnosis of imperforate hymen.

On postpartum day 1, pelvic MRI of the neonate showed normal uterine and cervical morphology, with an elongated and thickened vaginal wall, and no fluid collection (Figure 3). On postpartum day 2, the neonate underwent surgical exploration, which revealed a septum between the urethra and the vagina, with a completely covered vaginal orifice (imperforate hymen; Figure 4). A successful partial hymenectomy was performed.

On postpartum day 7, the surgical site was healing well without erythema or swelling. Subsequent ultrasound showed no fluid collection in the uterus or vagina.

## Discussion

Congenital imperforate hymen is a rare Müllerian duct anomaly resulting from the failure of the hymenal membrane to perforate during fetal development. It is one of the most common obstructive anomalies of the female reproductive tract, with an estimated incidence of 0.1%–0.5% in newborn females (4, 5). The condition often remains asymptomatic until adolescence, when patients typically present with primary amenorrhea, cyclic abdominal pain, and occasionally a palpable lower abdominal mass due to hematocolpos (accumulation of menstrual blood in the vagina) (5). In rare cases, it may be diagnosed prenatally or in infancy if associated with hydrocolpos (fluid accumulation in the vagina) due to maternal estrogen stimulation (4).

Prenatal ultrasound is an important tool for diagnosing fetal imperforate hymen. Ultrasound examination can reveal an anechoic area in the uterine cavity, as well as dilatation of the cervical canal extending to the vaginal orifice, with an anechoic area within (6). However, ultrasound findings can be influenced by factors such as fetal position and gestational age.

Fetal MRI, with its high resolution and ability to image in multiple planes and angles, is not affected by maternal or fetal positioning, and provides superior delineation of the detailed anatomy of the vagina and uterus for the diagnosis of imperforate hymen (7). In the present case, the fetal ultrasound was unable to clearly differentiate the bladder from the lesion, but the fetal abdominal MRI clearly visualized the dilated vagina and uterus, the distal vaginal obstruction (“reverse rabbit ear” sign), and the detailed anatomy of the uterus and vagina, providing a definitive diagnosis and guiding the treatment strategy.

The differential diagnosis of fetal imperforate hymen primarily includes transverse vaginal septum, vaginal atresia, and uterovaginal fluid accumulation (due to other causes) (8). On imaging, a transverse vaginal septum typically presents as fluid distension in the upper vaginal segment, with the septum located at a higher position, often near the cervix, distinguishing it from the distal obstruction seen in imperforate hymen (9). Vaginal atresia, on the other hand, is characterized by partial or complete absence of the vaginal canal, potentially associated with uterine developmental anomalies. Imaging reveals fluid accumulation in the upper vagina and uterus, with complete obstruction of the lower vaginal segment (10). Uterovaginal fluid accumulation due to other causes, such as cervical atresia or distal vaginal stenosis, requires precise localization of the obstruction via ultrasound or MRI (11). Imaging plays a pivotal role in differentiating these conditions, with imperforate hymen typically demonstrating obstruction at the distal vaginal level.

We described a case of a fetus with prenatal ultrasound-detected fetal pelvic cystic mass, which was further confirmed by MRI and underwent prenatal ultrasound-guided aspiration and drainage, followed by postnatal surgical treatment for hymenal atresia. *In utero* aspiration offers several potential benefits. Early decompression via drainage can alleviate pressure on fetal organs, potentially improving organ development and function (12). In cases of excessive fluid accumulation, which may lead to preterm labor, aspiration can help prolong gestation (13). Furthermore, early intervention may improve fetal survival rates and long-term prognosis in select cases. Finally, fluid obtained through aspiration allows for cytogenetic or biochemical analysis, aiding in the diagnosis of underlying conditions. It improves both fetal development and maternal well-being, making it a valuable intervention when performed by an experienced multidisciplinary team (14). In the present case, the patient had a strong desire for a vaginal delivery, but the enlarged fetal abdominal circumference could have complicated the delivery. Therefore, ultrasound-guided intrauterine aspiration was performed, which relieved the compression on the bladder and bowel, improving the patient's quality of life.

The postnatal management of imperforate hymen involves the prompt performance of a hymenotomy to establish an effective drainage pathway, prevent re-accumulation of fluid, alleviate pressure on surrounding organs, and avert long-term complications such as endometriosis, pelvic adhesions, or infertility (15, 16).

## Conclusion

The definitive diagnosis provided by the fetal MRI in this case was crucial for guiding the subsequent treatment plan. The ultrasound-guided intrauterine aspiration facilitated a successful vaginal delivery and the patient's postpartum recovery. The postnatal hymenectomy prevented the risk of future complications. Although the postoperative recovery was smooth, the short follow-up period limits the assessment of long-term prognosis, which will require closer long-term monitoring.

## Data Availability

The original contributions presented in the study are included in the article/Supplementary Material, further inquiries can be directed to the corresponding author.
